# Hybrid Repair of a True Brachiocephalic Artery Aneurysm: A Case Report

**DOI:** 10.3400/avd.cr.25-00104

**Published:** 2025-12-17

**Authors:** Takahiro Mizoguchi, Hiroshi Ito, Hiroshi Kurazumi, Masaya Takahashi, Yoshitaka Ikeda, Noriyasu Morikage, Kimikazu Hamano

**Affiliations:** 1 Division of Vascular Surgery, Department of Surgery and Clinical Science, Yamaguchi University Graduate School of Medicine, Ube, Yamaguchi, Japan; 2 Department of Cardiovascular Surgery, Yamaguchi Saiseikai Shimonoseki General Hospital, Shimonoseki, Yamaguchi, Japan; 3 Department of Cardiovascular Surgery, Kansai Medical University, Hirakata, Osaka, Japan

**Keywords:** brachiocephalic artery aneurysm, hybrid repair, endovascular repair

## Abstract

A 74-year-old man with hypertension and hyperuricemia was incidentally diagnosed with a 39-mm brachiocephalic artery aneurysm. Because of a sufficient proximal sealing zone, a hybrid repair was performed, consisting of a right common carotid–axillary bypass with a prosthetic graft, followed by endovascular exclusion using covered stents, a vascular plug, and coils. Completion angiography confirmed complete aneurysm exclusion without endoleak and satisfactory bypass flow. The postoperative course was uneventful. Computed tomography (CT) angiography demonstrated persistent aneurysm exclusion without endoleak and a patent bypass. At 12 months, plain CT confirmed no aneurysm enlargement.

## Introduction

Brachiocephalic artery aneurysms (BCAAs) are extremely uncommon, representing only 2%–5% of all aneurysms arising from the arch vessels.^1)^ In a cohort study of 73 patients with arch vessel aneurysms, Cury et al. noted that 63% were of degenerative origin, predominantly affecting men over the age of 60.^2)^ Other reported causes include traumatic injury, fibromuscular dysplasia, syphilitic infection, cystic medial necrosis, various forms of vasculitis, spread from adjacent infectious processes such as tuberculous lymphadenitis, and congenital factors.^2)^

The history of treating arch vessel aneurysms is longstanding. Historic reports include Halsted’s ligation of a subclavian aneurysm in 1892^3)^ and Muller’s case in 1935.^4)^ Kieffer et al. later classified BCAAs into Type A (distal to the origin), Type B (involving the origin), and Type C (extending into the ascending aorta).^5)^ Open surgical repair provides durable results but carries substantial morbidity, with operative mortality of up to 11% and respiratory complications requiring prolonged ventilatory support for longer than 5 days in 22%.^5)^ Endovascular repair is attractive but may be limited by inadequate landing zones and risk of compromising cerebral or vertebral circulation. Hybrid repair, combining extra-anatomic bypass with endovascular exclusion, has therefore emerged as a feasible, less invasive option in selected cases.^6–8)^ We herein report a case of a true Type A BCAA successfully treated by carotid–axillary bypass followed by endovascular exclusion.

## Case Report

**Figure 1:**
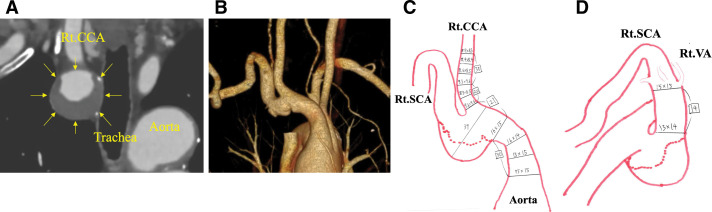
Fig. 1 Preoperative imaging. (**A**) Contrast-enhanced CT (coronal view) showing a 39-mm brachiocephalic artery aneurysm (yellow arrow). (**B**) Volume rendering reconstruction confirming the aneurysm arising from the brachiocephalic trunk. (**C**) Measurement at the proximal sealing zone of the ascending aorta (values in mm). (**D**) Measurement at the level of the right SCA origin (values in mm). Rt. CCA: right common carotid artery; Rt. SCA: right subclavian artery; Rt. VA: right vertebral artery; CT: computed tomography

A 74-year-old man with hypertension and hyperuricemia was incidentally found to have a saccular aneurysm of the brachiocephalic artery (BCA) measuring 39 mm on contrast-enhanced computed tomography (CT) (**Fig. 1A**). Three-dimensional reconstruction confirmed the aneurysm arising from the brachiocephalic trunk (**Fig. 1B**). On preoperative sizing (**Fig. 1C**), the 3-cm distance between the BCA origin and the aneurysm allowed for an adequate proximal sealing zone. At the level of the right subclavian artery (SCA) origin (**Fig. 1D**), the distance from the subclavian to the vertebral artery (VA) was only 1.4 cm, but precise embolization with a coil-in-plug strategy was considered sufficient to control backflow while preserving VA flow. Preoperative magnetic resonance angiography (MRA) demonstrated sufficient intracranial communication through the vertebrobasilar system, and adequate perfusion from the left VA to the basilar artery. In the event of stent-graft migration or endoleak, secondary open surgical repair was considered as a bailout option.

### Operative details

Under general anesthesia with endotracheal intubation, the patient was placed supine. Five-centimeter incisions were made at the right neck and infraclavicular region to expose the right common carotid artery (CCA) and right axillary artery (AxA).

A 7-mm ringed polytetrafluoroethylene (PTFE) graft (Propaten; W. L. Gore & Associates, Flagstaff, AZ, USA) was tunneled subclavicularly. During tunneling, a branch of the internal jugular vein was avulsed and repaired with 5-0 Prolene (Ethicon, Somerville, NJ, USA), Z-suture. A side-to-end anastomosis was performed proximally to the CCA and distally to the AxA, using 5-0 CV continuous sutures (W. L. Gore & Associates).

**Figure 2:**
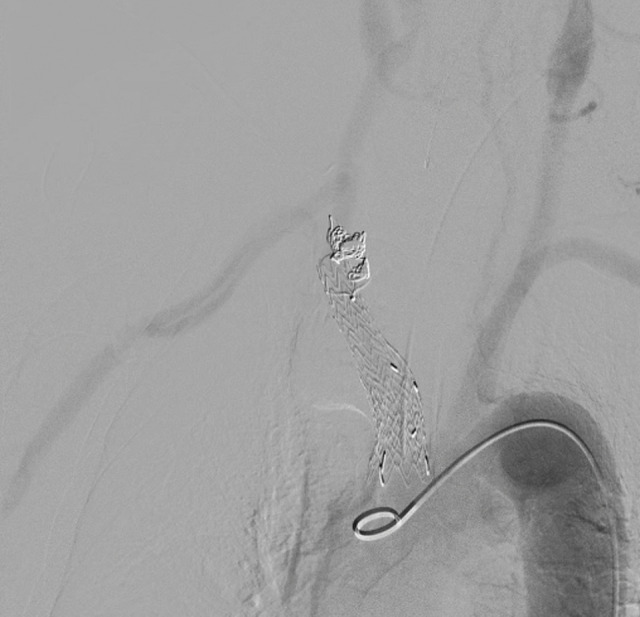
Fig. 2 Completion angiography after hybrid repair showing complete exclusion of the aneurysm without endoleak and satisfactory flow through the carotid–axillary bypass graft.

For endovascular exclusion, the right CCA was punctured and a 16-Fr DrySeal sheath (W. L. Gore & Associates) was advanced. An Excluder iliac extender (PLL161207; W. L. Gore & Associates) was deployed from the BCA into the right CCA, followed by an Excluder aortic extender (PLA230300J; W. L. Gore & Associates) at the BCA origin. Both were post-dilated with a Reliant balloon catheter, 46 mm (Medtronic, Minneapolis, MN, USA). For subclavian exclusion, a 16-mm Amplatzer vascular plug (Abbott, Abbott Park, IL, USA) was deployed at the right subclavian origin, supplemented with coil-embolized (POD coil; Penumbra, Alameda, CA, USA), enabling precise occlusion while preserving the right VA. Completion angiography confirmed successful aneurysm exclusion without endoleak and satisfactory bypass flow (**Fig. 2**). Operative time was 3 h 42 min; blood loss 830 mL; contrast volume 140 mL; fluoroscopy time 20 min 40 s; air kerma 902 mGy.

### Postoperative course

**Figure 3:**
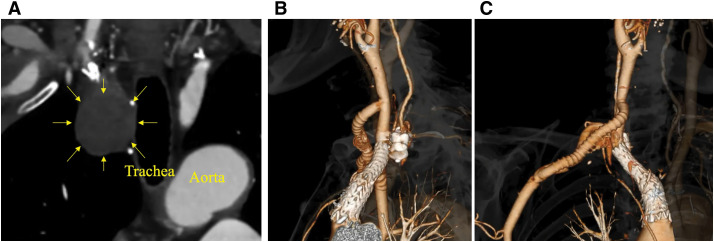
Fig. 3 Postoperative follow-up imaging. (**A**) Contrast-enhanced CT (coronal view, 1 month) demonstrating exclusion of the aneurysm. (**B**) Volume rendering reconstruction in right anterior oblique projection at 1 month showing no endoleak. (**C**) Volume rendering reconstruction in left anterior oblique projection at 1 month, confirming a bypass patency. CT: computed tomography

The postoperative course was uneventful, and the patient was discharged on postoperative day (POD) 9. One-month CT angiography demonstrated patent bypass and right VA, and no endoleak (**Figs. 3A**–**3C**). At 12 months, plain CT confirmed the absence of aneurysm enlargement.

## Discussion

Indications for repair of BCAAs generally include symptomatic presentation, aneurysm diameter greater than 30 mm, or rapid enlargement.^5)^ Traditional open surgical repair provides durable results but may require sternotomy or cardiopulmonary bypass, with considerable morbidity. Kieffer et al. reported an operative mortality of 11% and respiratory failure requiring prolonged ventilatory support in 22% of cases.^5)^ Purely endovascular approaches are often limited by inadequate landing zones and the risk of compromising cerebral or vertebral circulation.

Hybrid repair has therefore been increasingly adopted as a less invasive alternative, combining surgical bypass for cerebral and upper extremity perfusion with endovascular exclusion of the aneurysm. Favorable results have been reported by Angiletta et al. and Resch et al., although perioperative stroke remains a concern.^7,8)^ In the present case, a 3-cm proximal sealing zone enabled safe stent-graft placement, while a carotid–axillary bypass secured antegrade flow. Adjunctive embolization of the right SCA with a plug and coils allowed preservation of vertebral perfusion and prevented type II endoleak, demonstrating an important technical advantage of the hybrid strategy. The combined use of a plug and coils not only facilitated accurate deployment but also reinforced the occlusive effect, enabling successful exclusion even in anatomically constrained settings such as the present case, where the distance from the right SCA origin to the right VA was 14 mm, a length that did not provide ample margin. Furthermore, from a technical standpoint, in procedures requiring tunneling of prosthetic grafts, as in our case, one must exercise particular caution. Unlike open sternotomy, the limited operative field can predispose to fatal bleeding should inadvertent vascular injury occur.

Limitations of our case include the relatively short follow-up of only 12 months. Long-term durability of hybrid repair for BCAAs remains uncertain, given the rarity of reported cases and lack of extended follow-up. Nevertheless, our case illustrates that hybrid repair is a safe and feasible option for anatomically favorable true Type A aneurysms, particularly in elderly or comorbid patients. Notably, even in the setting of infectious aneurysms, successful hybrid repair has been reported by Go et al., underscoring the versatility of this approach in complex clinical scenarios.^9)^ On the other hand, Hu et al. reported successful treatment of a pseudoaneurysm near the innominate artery bifurcation using kissing covered stents.^10)^ While this approach may be much less invasive and effective in selected cases, the risk of gutter endoleak and concerns regarding long-term patency render it less reliable compared with hybrid repair.

## Conclusion

Hybrid repair—combining carotid–axillary bypass with endovascular exclusion—provides a safe, less invasive, and effective option for selected Type A BCAAs with favorable anatomy.
